# Energy and Macronutrients Intakes among Childbearing Age Women Living in the Urban Area of Morocco: A Cross-Sectional Study

**DOI:** 10.1155/2020/2685809

**Published:** 2020-09-11

**Authors:** Fatima Barich, Fatima Zahra Laamiri, Slimane Mehdad, Souad Benaich, Anass Rami, Mohamed Idrissi, Chaimae Serbouti, Houria Lahmame, Hasnae Benkirane, Manelle Rjimati, Amina Barkat, El Arbi Rjimati, Hassan Aguenaou

**Affiliations:** ^1^Joint Unit of Nutrition and Food Research, CNESTEN–Ibn Tofäιl University–URAC 39, Regional Designated Center for Nutrition (AFRA/IAEA), Rabat, Morocco; ^2^Higher Institute of Nursing and Medical Professions, Rabat, Morocco; ^3^Mother and Child Couple Health and Nutrition Research Team, FMP de Rabat, Mohammed V University, Rabat, Morocco; ^4^Health Sciences and Technology Laboratory, Higher Institute of Health Sciences of Settat, Hassan First University, Settat, Morocco; ^5^Physiology and Physiopathology Research Team, Faculty of Sciences, Mohammed V University, Rabat, Morocco

## Abstract

Over the last few decades, there have been significant dietary and lifestyle changes worldwide. In Morocco, these changes have led to serious nutritional disorders and increased risk of morbidity and mortality particularly among vulnerable groups such as women of childbearing age. We aimed to assess the average daily energy and macronutrient intakes and to investigate their association with socioeconomic factors and weight status among women aged 19–49 years in urban areas. A total of 542 women attending public health centers were recruited. Socioeconomic and demographic data were collected using a questionnaire. Anthropometric measurements were taken using standardized equipment. Food consumption data were obtained through the 24-hour dietary recall method, and the macronutrient composition of foods was estimated based on the Moroccan food composition table and the Nutrilog software. The average daily energy intake among the study population was 1591 kcal, composed of 56% from carbohydrates, 28% from fats, and 16% from protein. Reported energy intake by the majority of women (81.5%) was lower than recommended daily allowances for energy. There was a significant positive correlation between educational level and energy (*p*=0.001), carbohydrates (*p*=0.001), proteins (*p*=0.004), and fats intakes (*p*=0.032), respectively. A significant negative association of household size with protein intakes was also observed (*p*=0.034). Carbohydrates, proteins, and fats intakes tended to decrease; however, these associations were not statistically significant. Further studies and appropriate interventions are needed to address the trends in energy and macronutrients intakes in the development of policy initiatives aimed at nutrition education and chronic disease prevention among childbearing age women.

## 1. Introduction

Malnutrition is defined as deficiencies, excesses, or imbalances in individuals' intake of energy and/or nutrients [1]. A balanced diet can be achieved when the total energy intake is covered at 10 to 15% from protein, 15 to 30% from fat, and 50–55% from carbohydrate [2, 3]. Malnutrition is characterized by an inadequate intake of protein, energy, or micronutrients, aggravated by frequent infections or diseases [4, 5]. It is currently the most serious nutritional problem in many countries in Asia, Latin America, the Middle East, and Africa [6]. Malnutrition increases health care expenditure and reduces productivity and economic growth, which can result in a cycle of poverty and poor health [1, 7]. Women, infants, children, and adolescents are particularly most exposed to malnutrition [1].

Paradoxically, many parts of the world are currently exposed to overweight and obesity epidemic, with over 39% of adults being overweight globally especially due to an energy imbalance that results from increased caloric intake and reduced energy expenditure [8]. Consequently, the burden of noncommunicable diseases like cardiovascular diseases, diabetes, musculoskeletal disorder, and some cancers is increasing [8–10]. The prevalence of these diseases in developing countries is increasing at an alarming rate, particularly in urban areas, and remains one of the major public health concerns [11, 12].

Over the last few decades, the Moroccan population has been facing rapid dietary and lifestyle changes. Changes have led to serious nutritional disorders and increased prevalence of noncommunicable diseases (NCD) in all age groups. For instance, the survey conducted in 2018 reported a high prevalence of hypertension, diabetes, and hypercholesterolemia among Moroccan women aged 18 years and over (29.8%, 12.6%, and 14.0%, respectively) [13]. These NCDs are often associated with obesity which can also lead to many other health complications including cardiovascular and respiratory disorders [8, 10]. Also, obesity has recently reached unprecedented prevalence (29.0%) among women [13], which could significantly increase the risk of morbidity and mortality in this population segment, particularly among women of childbearing age.

As all Moroccan regions, Rabat-Salé-Kenitra region has been experiencing a dramatic increase in obesity prevalence rates among women during the past two decades [14]. However, to our knowledge, there is no information on energy and macronutrient intakes as important factors contributing to weight gain particularly among women of childbearing age living in this region. Thus, our study aimed to assess the average daily energy and macronutrient intakes and to investigate their association with nutritional status and socioeconomic factors among a sample of women aged 19–49 years from urban areas surrounding Rabat, the capital city of Morocco.

## 2. Materials and Methods

### 2.1. Population

This study is a descriptive cross-sectional survey. It was carried out on healthy women recruited from public health centers located in Rabat-Salé-Kenitra region. It included women aged 19–49 years who were not under any permanent medical treatment. Pregnant and lactating women, women suffering from mental illnesses, and those who participated in the pilot study were excluded from the current study.

### 2.2. Site of the Study

The study took place in urban areas of Rabat, Salé, and Skhirat-Témara prefectures known for their high rates of obesity (ENSF, 2003-2004) [14].

### 2.3. Selection of Health Centers

The women were recruited from 21 selected urban health centers based on the following criteria: accessibility of our field team and high participation of women to cover the number required for the age group under study.

### 2.4. Sample

The study included 542 Moroccan childbearing age women aged between 19 and 49 years who visited the health centers during the period of data collection. Only women who met the inclusion criteria and provided their consent to participate in the survey were recruited.

To calculate the sample size, we used the formula developed by Cochran (1977) [15] and Ardilly (2006) [16]:

(1) $$\begin{array}{c} n=t2\times p\times \frac{\left(1-p\right)}{m2}, \end{array}$$

where *t* is the 95% confidence level, *p* is the estimated prevalence of the obese population, and *m* is the error margin (set at 5%).

The estimated prevalence of obesity in the targeted region was 14.4% with an error margin of 5% [14]. Thus, 189 subjects were considered necessary for inclusion to obtain statistically representative data. The total number of women who met the inclusion criteria was 542.

### 2.5. Anthropometric Parameters

The anthropometric parameters of each participant were measured according to the WHO standard procedures [17]. Body weight was measured to the nearest of 0.1 kg using a scale (Seca 750) with a minimum of clothing and no shoes. The height was measured to the nearest of 0.1 cm using a stadiometer (Seca). Body mass index (BMI) was calculated as the ratio of weight in kg to square height in m^2^. Women were classified into four groups according to BMI [17, 18]:

- Women with underweight: BMI < 18.5 kg/m^2^
- Women in a normal weight range: 18.5 kg/m^2^ ≤ BMI ≤ 24.99 kg/m^2^
- Overweight women: 25 kg/m^2^ ≤ BMI ≤ 29.99 kg/m^2^
- Obese women: BMI ≥ 30 Kg/m^2^

Waist circumference was measured midway between the lower edge of the last palpable rib and the top of the iliac crest [19]. The hip circumference was measured at the widest point on the buttocks of the study participants. For abdominal obesity, waist-to-hip ratio (WHR) was obtained by dividing the average waist circumference by the average hip circumference. Women with WHR ≥ 0.85 were classified as abdominally obese [17].

### 2.6. Data Collection

#### 2.6.1. Socioeconomic and Demographic Factors

The data on socioeconomic and demographic factors were collected at the beginning of the study through direct interviews. We used an adequate questionnaire that was adapted from other ones used nationally for a similar purpose [14].

The collected information included women's education level, marital status, occupation, occupation of the household head, number of children, household size, household monthly global expenses, and food expenses.

#### 2.6.2. Dietary Intake Assessment

*(1) The 24-Hour Recall Questionnaire*. Food intake data were collected using the 24-hour recall questionnaire, a rapid and inexpensive method that could be used for nutrition surveys in large groups [20].

This questionnaire includes information related to quantity and nature of food eaten (bread, tomato, tea, water, etc.) for breakfast, lunch, and dinner as well as midmorning and midafternoon snacks.

In this study, the multipass approach was used to validate the 24-hour recall questionnaire. The principle of this method consists of 5 steps [21, 22]:

- Quick list: to collect a list of foods consumed the previous day.
- Forgotten foods list: to collect foods that may have been forgotten during the quick list. Questions probe for foods by categories: nonalcoholic beverages, alcoholic beverages, sweets, savory snacks, fruits, vegetables, cheese, bread and rolls, and other foods.
- Time and occasion: to collect time and name of eating occasion for each food, used to sort foods chronologically and group into eating occasions.
- Detail and review: to collect a detailed description of each food consumed, including the amount eaten and additions to the food, and also to review eating occasions and times between occasions to elicit forgotten foods.
- Final review: to collect additional foods not remembered earlier.

Women were asked to provide all information about foods and drinks consumed for two separated days using visual aids to approximate the serving sizes of various foods: a photo manual (SU.VI.MAX) for estimating consumed portions of food and drink [23] and a food book to estimate the amount of food consumed based on food and typical preparations of the Moroccan population [24].

The quantities consumed were converted using the Food Quantification Table, available in the corresponding book, then entered into the Nutrilog Food Information Software (SAS, version: 2.31, with Moroccan Food Composition Table), which was used to determine nutrients and vitamins content of each food. At the same time, all details on vitamin and mineral supplements consumed during the investigation period were noted and the average macronutrient intake over two days was reported to estimate the exact quantities permitting comparison with recommended dietary intakes.

*(2) Nutrilog Software*. The Nutrilog software (version 2.31) is a professional nutrition tool based on nutritional references of the ANSES (French national agency of health safety of nutrition, environment, and employment) [25]. It provides information on the individual food profile. The Moroccan food composition table has been integrated into the software database containing the CIQUAL food table, France 2012; USD SR24; USA 201 [25] provided by the supplier. It is worth mentioning that the Moroccan food composition table is the first table developed and includes 424 foods consumed in our country.

### 2.7. Statistical Analysis

The data matrix compiled by the Nutrilog software, combined with socioeconomic and demographic data, was analyzed with statistical software for the social sciences (SPSS version 13.0). The normality of the distribution of anthropometric and nutritional variables was assessed using the Kolmogorov-Smirnov (KS) test [26]. Continuous variables with Gaussian distribution were expressed as mean ± standard deviation, while non-Gaussian distribution variables were expressed as median (interquartile range). Categorical variables were expressed as counts and percentages. We used the Chi-square test to compare energy intake between different age groups. Pearson correlation test was used to examine the association of energy and macronutrients intakes with demographic and socioeconomic factors. Finally, the Kruskal-Wallis test was used to compare macronutrients and energy intakes among BMI groups. A value of *p* < 0.05 was considered significant.

### 2.8. Ethical Considerations

The study protocol was approved by the Ethics Board of the Faculty of Medicine and Pharmacy, Mohammed University in Rabat, Morocco (Ethical Approval number 69 delivered on 31 January 2017). Before data collection, invited participants were informed about the study objectives and methods, and both oral and written consent were obtained from all who were recruited.

## 3. Results

### 3.1. Demographic and Socioeconomic Characteristics

The demographic and socioeconomic characteristics of the study population are presented in Table 1. The majority of women had Arab ethnic background (77.1%). Over two-thirds were without job (70.7%), 17.2% were illiterate, and more than a third of women (38.4%) had a secondary education level. Half of women (50%) had 1 to 2 children while most families (72.0%) were composed of 4 to 7 people. The monthly expenditure was less than US$ 366 in 56.5% of the study population, and more than 66% spent less than US$ 272.32 per month for food.

### 3.2. Anthropometric Parameters

**Figure 1:**
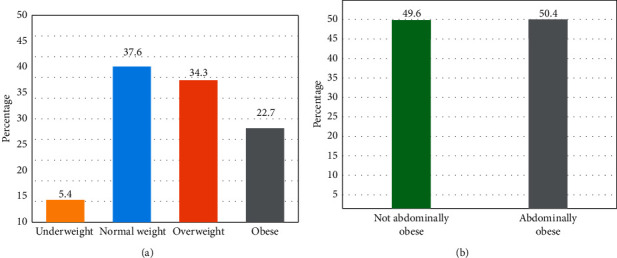
Prevalence of overall obesity, overweight (a), and abdominal obesity (b) among the study population. (a) BMI groups. (b) WHR groups.

The anthropometric parameters of the participants are presented in Table 2. The average weight and height were 67.84 ± 13.64 kg and 1.60 ± 0.06 m, respectively. Mean values for BMI, waist circumference (WC), hip circumference (HC), and waist-to-hip ratio (WHR) were 26.35 ± 5.21 kg/m^2^, 87.60 ± 12.98 cm, 68 ± 12.98 cm, and 0.86 ± 0.08 cm, respectively. The prevalence of overweight and obesity was 34.3% and 22.7%, respectively, while more than 50% of women had abdominal obesity (Figure 1).

### 3.3. Energy and Macronutrients Intakes

Table 3 illustrates the energy and macronutrient intakes among the study population. The average daily energy intake was 1591.2 ± 664.4 kcal, 56% from carbohydrates, 28% from fats, and 16% from proteins. The majority of women (81.5%) reported an energy intake lower than recommended daily allowances for energy, with a significant difference between age groups (*p*=0.043).

### 3.4. Association of Macronutrients and Energy Intakes with Demographic and Socioeconomic Variables

Table 4 shows the correlation between energy, macronutrients, and the socioeconomic status of participants. The analysis showed a significant positive correlation between educational level and each of energy (*p*=0.001), carbohydrates (*p*=0.001), protein (*p*=0.004), and fat intakes (*p*=0.032). A negative association of household size with macronutrients and energy intakes was also observed in the study population. This association was statistically significant for protein intake (*p*=0.034) and reached borderline statistical significance for energy and fats intakes (*p*=0.080 and *p*=0.066, respectively). Carbohydrates, proteins, and fats intakes tended to decrease as age increased; however, these associations were not statistically significant. Similarly, the correlations of macronutrients and energy intakes with other socioeconomic and demographic variables were not statistically significant.

### 3.5. Association of Energy and Macronutrients Intakes with Nutritional Status

Both underweight and obese women had a slightly higher average of daily energy and macronutrient intake compared to normal weight and overweight groups, but the difference was not statistically significant (Table 5).

## 4. Discussion

The present study aimed to provide information on the nutritional status of childbearing age women living in urban areas of the Rabat-Salé-Kenitra region. To our knowledge, it was the first time to estimate macronutrient and energy intakes and investigate their association with weight status and demographic and socioeconomic factors among this age group at both regional and national level, using three dietary assessment tools (food survey (24 h recall), Nutrilog software, and Moroccan food composition table).

Our results showed that 34.3% and 22.7% of women were overweight and obese, respectively. These prevalence remains high compared to those reported in a previous national survey published in 2005 [14]. Similarly, obesity prevalence was greater than that reported in 2016 (19.97%) by the High Commission for Planning (HCP) [27] but remains lower than those observed in other regions in Morocco [28, 29] as well as in Tunisia [30]. Furthermore, based on the WHO standards [19], we found that 50.4% of women were abdominally obese. Our results are consistent with those reported in a recent similar study indicating a high prevalence of abdominal obesity in women of childbearing age [31] and confirm the rising trends of overall and abdominal obesity in the Moroccan population, particularly among women of childbearing age.

Moreover, as excess body weight is recognized as an important risk factor for many noncommunicable diseases [8, 9, 32], our findings confirm also the possible role of overweight and obesity in the rapidly growing burden of these diseases whose economic cost represents about 14% of the total health expenditure in Morocco [33].

Despite all the efforts made at the national, regional, and international levels, malnutrition remains a major health problem in the EMRO region and its health consequences are too important to be neglected [34]. Many studies in Morocco have been conducted to determine the magnitude of malnutrition in different age groups such as infants and preschool children, but few of them explored its impact on women of childbearing age. In this study, the majority of women (81.5%) did not meet the daily energy intake (2140 kcal) recommended by the Food and Agriculture Organization of the United Nations (FAO) [35]. Assuming that the estimates of intake are correct, as most of the participants (57%) were either overweight or obese, this is likely due to reducing dietary energy intake or caloric restriction in order to lose weight or prevent excess weight gain. Moreover, the low daily energy intake could be attributed to using the 24-hour dietary recall which may lead to underestimating the actual consumption of nutrients and foods [36], especially among overweight or obese individuals who tend to underreport their energy intake [37, 38]. Underreporting also remains one of the most common types of errors, known as “flat slope syndrome” where people generally tend to increase their intake when it is low and decrease it when it is high [39]. Other factors could influence the accuracy of self-reported nutrient and energy intake such as sex and body image perception. Studies have shown that energy misreporting is more often associated with female gender [40–42], as well as with body image dissatisfaction [40]. Likewise, some researchers have suggested that this double burden of malnutrition could also be explained by the possibility that the biological characteristics of these individuals make them more predisposed to a rapid increase in adiposity [43]. Our results support the hypothesis that using a combination of methods, such as the 24-hour dietary recall and biomarker levels, may provide more accurate estimates of the dietary intake than that of individual methods [44]. However, further research is required to assess dietary intake and investigate the impact of energy under- and overreporting on diet-obesity relationships in this population.

Similarly, the daily protein intake of our study population (61.5 ± 32.1 g) was lower than that found in similar age groups from South Africa and Kuwait (125.3 g and 67.4 ± 2.3 g, respectively) [45, 46], but it provided around 16% of energy intake, which is in line with the 15% recommended by the international community for women of childbearing age [47, 48]. Moreover, our results are consistent with those found by Zaghloul et al. among Kuwaiti women [46].

Protein intake was dominated by vegetable proteins that accounted for more than two-thirds of the daily protein intake in our study population. Although it is difficult to compare our results with those found by other studies because of their great variability. Our findings are in agreement with those reported by Steyn et al. who found a low contribution of proteins to energy intake in Kenyan women, particularly for animal protein intake [45]. However, these findings differ from those of other authors, such as Souza et al. who observed a higher intake of animal proteins among Brazilian women [49]. Given the physiological role of animal proteins and human body needs [50], the imbalance between nutrient requirements (50% of animal proteins-50% of plant proteins) [10] and nutrient intake observed in our study could have negative effects on women's health. Indeed, the predominance of vegetable proteins may prevent the human body from covering its essential amino acid needs [10, 51]. It may also inhibit the absorption of some micronutrients such as hemic iron and zinc [52]. Thus, despite the dietary diversity and food resources available in the Rabat-Salé-Kenitra region, including fisheries resources [53, 54], there is a need to improve the quality and quantity of protein-rich foods to have a healthy and balanced diet among women of reproductive age.

Regarding carbohydrates, the estimated daily intake was 218.89 ± 89.05 g and it accounted for an average of 56 % of the mean daily energy intake. This amount exceeded both the recommended daily carbohydrates intake (130 g/d) and its contribution to energy requirements (50–55% from carbohydrates) [4]. Our findings are consistent with the FAO's claim that the main source of energy for most Africans, Asians, and South Americans is carbohydrates [35]. These findings also showed that current dietary patterns of our study population are characterized by elevated carbohydrates intake which may cause a loss of the diet palatability by an excessive reduction of lipid consumption [51] and that increases the risk of developing obesity and related chronic diseases [55]. Thus, our study suggests that increased education about a balanced diet and healthy eating practices is necessary to ensure the recommended percentage contribution of calories from macronutrients, particularly carbohydrate calories that should not exceed 55% of total energy intake [51].

The average fat intake was 52.2 ± 34.8 g and its contribution to the average daily energy intake was 28%. This contribution corresponds to the internationally recommended rate (15–30%) [47, 48]. The saturated fat intake expressed as a percentage of total energy intake (E) was 7.4 ± 3.8 < 10%. These results are consistent with the WHO recommendations. The saturated fatty acids contribution to the daily energy intake of our study population was also lower than that of a similar study conducted among Kenyan women living in urban areas (12%) [45]. Nevertheless, the intake of polyunsaturated fats observed in our study was 4.7 ± 2.9%. It remains relatively low to have significant health benefits, since the reduced level of this nutrient is associated with an increased risk of cardiovascular disease [56] and high levels of LDL cholesterol [57].

In the current study, a significant positive association of education level with energy, carbohydrate, protein, and fat intakes was observed. Our results are in agreement with those reported by Fryar et al. who observed a similar correlation between energy intake and education level among American women of Mexican origin [58], while they are not consistent with those of a study conducted among Kuwaiti women [46]. Overall, this correlation may be due, at least in part, to improved socioeconomic status and easy access to energy-dense foods among more educated women in Morocco.

Our results revealed a negative correlation between household size and protein intake, which confirm results reported by other authors [59, 60]. Although the relationship between household size and socioeconomic status was not examined in this study, the observed correlation may be a result of less advantaged socioeconomic living conditions of larger family sizes.

Regarding the association of weight status with energy and macronutrient intakes, we found no statistically significant difference between various BMI groups. In agreement with our results, a previous study has shown an inconsistent association between energy and macronutrient intakes and increased BMI among Thai urban women [61]. However, many other studies demonstrated direct positive associations of energy and sugars with excess body weight [62, 63]. As it is widely recognized that body weight gain occurs when energy intake exceeds energy expenditure, our findings could be explained by the relatively low daily energy intake among the study population.

The present study has certain limitations. First, our findings are based on the 24-hour dietary recall method and the associated underreporting of energy and macronutrient intakes is a well-documented problem of such dietary assessment tools. Second, the cross-sectional design of the study makes it impossible to draw causal relationships between different variables. However, this study provides data that can help advance knowledge about the association of dietary variables with weight status and socioeconomic factors and show to researchers and nutrition policymakers the importance of addressing macronutrient and energy intake in nutritional status research, which is very relevant in light of the current global obesity epidemic.

In conclusion, most women did not meet the recommended daily energy intake. The daily macronutrient and energy intakes were positively associated with educational level. In contrast, there was a negative association of household size and age with carbohydrate, protein, and fat intakes. Despite the low average energy intake, more than half (57%) of the study population was either overweight or obese. These results emphasize the need for interventions to improve the dietary intake in nutritionally vulnerable women. Considering the role of overweight and obesity as major risk factors for many chronic diseases [10, 64], our findings also suggest to put in place appropriate strategies to prevent and control the double burden of malnutrition and noncommunicable diseases which remain the leading causes of death (78%) in Morocco [65]. However, further studies are warranted to address the double burden of malnutrition and its risk factors in women of various socioeconomic groups living in urban areas.

## Figures and Tables

**Table 1:** Demographic and socioeconomic characteristics of the study population.

Variables	*n*	%	95% CI
*Age groups (years)*			
19 to 29	240	44.2	40.4–48.5
30 to 40	203	37.5	33.4–41.3
41 and more	99	18.3	15.1–21.6

*Ethnic background*			
Arab	418	77.1	73.8–80.6
Amazigh	96	17.7	14.6–20.7
Sahraoui	12	2.2	1.1–3.5
Rifian	9	1.7	0.7–2.8
Jebli	7	1.3	0.2–2.4

*Level of education*			
Illiterate	93	17.2	14.0–20.3
Primary	113	20.8	17.2–24.5
Secondary	208	38.4	34.3–42.8
Higher education	128	23.6	19.9–27.3

*Marital status*			
Single	111	20.5	17.3–24.2
Married	405	74.7	70.8–78.2
Divorced	22	4.1	2.6–5.5
Widowed	4	0.7	0.2–1.5

*Occupation of women*			
Without job	383	70.7	66.6–74.5
Student	52	9.6	7.4–12.2
With job	107	19.7	16.6–23.1

*Occupation of the household head*			
Without job	16	3.0	1.7–4.4
With job	516	95.2	93.4–96.9
Retired	10	1.8	0.7–3.1

*Number of children*			
No child	133	24.5	20.8–28.6
1 to 2 children	271	50.0	45.6–53.7
3 or more	138	25.5	22.1–29.3

*Monthly expenditure (US$)*			
Lower than 122	4	0.7	0.2–1.7
122 to 195	39	7.2	5.0–9.4
196 to 244	144	26.6	22.9–30.4
244 to 366	119	22.0	18.5–25.3
Higher than 366	124	22.9	19.2–26.4
Not known	112	20.7	17.3–24

*Monthly expenditure for food (US$)*			
Lower than 65.36	21	3.9	2.4–5.5
65.47 to 98.03	55	10.1	7.7–12.7
98.14 to 130.71	56	10.3	7.8–12.7
130.82 to 174.28	132	24.4	21.0–28
174.39 to 272.32	97	17.9	14.8–21.4
Higher than 272.32	69	12.7	10.1–15.7
Not known	112	20.7	17.3–24.0

*Household size*			
<4 people	107	19.7	16.6–23.2
4 to 7 people	390	72.0	68.1–75.6
>7 people	45	8.3	6.1–10.7

*Note.* Values are expressed in count and percentage, CI: 95% confidence interval.

**Table 2:** Age and anthropometric characteristics of the study population.

	Mean	Error standard	95% confidence interval	Minimum-maximum
Age (years)	31.96 ± 8.42	0.36	—	19–49
Weight (kg)	67.84 ± 13.64	0.59	—	39–128
Height (cm)	1.60 ± 0.06	0.01	—	1.42–1.78
BMI (kg/m^2^)	26.35 ± 5.21	0.22	—	15.73–42.06
*BMI group*				
Underweight group	29 (5.4%)	1.0	3.7–7.4	
Normal group	204 (37.6%)	2.1	33.4–41.7	
Overweight group	186 (34.3%)	2.1	30.3–38.4	
Obesity group	123 (22.7%)	1.8	19.2–26.4	
WC (cm)	87.60 ± 12.98	0.56	—	57–128
HC (cm)	101.68 ± 12.98	0.47	—	43–135
WHR	0.86 ± 0.08	0.01	—	0.64–1.52

*Notes*. Values are expressed as mean ± standard deviation or number and percent. For groups with a body mass index (BMI): underweight, BMI ≤ 18.5 kg/m^2^; normal BMI = 18.5 to < 25 kg m^2^; overweight BMI = 25 to < 30 kg/m^2^; obese BMI ≥ 30 kg/m^2^. WC: waist size < 80 cm; HC: hip circumference < 88 cm; WHR: waist/hips ratio ≥ 0.85 cm.

**Table 3:** Average daily energy and macronutrients intakes by age groups.

Variables	19 to 29 years (*n* = 240)	30 to 40 years (*n* = 203)	41 years and more (*n* = 99)	All (*n* = 542)
Energy (kcal/d)^*α*^	1648.4 ± 740.7	1543.9 ± 587.3	1549.8 ± 611.3	1591.2 ± 664.4
Underconsumption compared to EER^ß^	184 (76.7)	175 (86.2)	83 (83.8)	442 (81.5)
Overconsumption relative to EER^ß^	56 (23.3)	28 (13.8)	16 (16.2)	100 (18.5)
Protein (g)^*α*^	63.8 ± 34.2	59.6 ± 31.0	59.6 ± 28.7	61.5 ± 32.1
Animal protein (g)^ɣ^	13.0 [3.7–26.0]	11.1 [2.7–26.2]	9.3 [2.0–25.9]	11.7 [3.2–25.9]
Vegetable protein (g)^ɣ^	40.4 [27.9–58.9]	36.4 [25.3–52.4]	40.4 [26.4–49.7]	38.9 [26.5–55.1]
% of protein in energy intake^*α*^	15.7 ± 4.4	15.4 ± 4.8	15.6 ± 4.3	15.6 ± 4.5
Carbohydrates (g)^*α*^	225.0 ± 99.7	211.0 ± 77.0	220.0 ± 84.1	218.8 ± 89.0
% of carbohydrates in energy intake^*α*^	55.8 ± 10.6	55.7 ± 10.7	57.6 ± 10.5	56.1 ± 10.6
Fat (g)^*α*^	54.7 ± 36.9	51.3 ± 33.9	47.8 ± 31.2	52.1 ± 34.8
% of fat in energy intake^*α*^	28.3 ± 10.0	28.9 ± 10.9	26.7 ± 10.6	28.2 ± 10.4
Saturated fatty acids (g)^ß^	26.5 [20.1–33.6]	25.0 [19.60–34.90]	25.0 [19.4–34.2]	7.4 ± 6.8
% of fatty acids in energy intake^ɣ^	6.9 [5.1–9.4]	6.81 [5.2–9.10]	6.6 [4.9–8.2]	7.4 ± 3.8
Polyunsaturated fatty acids (g)^ɣ^	15.6 [11.3–23.5]	14.0 [10.8–20.9]	15.0 [11.6–21.7]	4.7 ± 4
% of polyunsaturated fat in energy intake^ɣ^	4.1 [2.9–5.8]	3.8 [2.9–5.5]	3.9 [2.6–5.3]	4.7 ± 2.9

*Note*. *α* values are expressed as mean ± standard deviation; ß values are expressed as count and percent; ɣ values are expressed as median (quartiles). EER: estimated energy requirement.

**Table 4:** Association of energy and macronutrients intakes with weight status and demographic and socioeconomic factors among the study population.

	Energy (kcal/d)		Carbohydrates (g)		Protein (g)		Fat (g)	
	*r*	*p*	*r*	*p*	*r*	*p*	*r*	*p*
Age	0.069	0.106	−0.055	0.200	−0.058	0.177	−0.065	0.133
Ethnic background	−0.013	0.757	−0.035	0.414	−0.016	0.714	0.007	0.879
Education level	0.138^∗^^∗^	0.001	0.148^∗^^∗^	0.001	0.125^∗^	0.004	0.092^∗^	0.032
Marital status	−0.021	0.623	−0.038	0.374	0.019	0.651	−0.018	0.682
Women's occupation	0.009	0.828	0.006	0.897	0.043	0.315	0.006	0.883
Size of household	−0.075	0.080	−0.046	0.286	−0.091^∗^	0.034	−0.079	0.066
Monthly expenditure	−0.014	0.741	0.040	0.352	0.038	0.378	−0.036	0.402
Monthly expenditure for food	−0.005	0.915	0.055	0.200	−0.017	0.698	−0.042	0.335

*Note.α* values are expressed as mean ± standard deviation; ß values are expressed as count and percent; ɣ values are expressed as median (quartiles). EER: estimated energy requirement.

**Table 5:** Association of energy and macronutrients intakes with nutritional status.

Variables	Underweight (*n* = 29)	Normal weight (*n* = 204)	Overweight (*n* = 186)	Obese (*n* = 123)	*p* value^∗^
Energy (kcal/d)^*α*^	1744.0 [1308.5–2183]	1497.0 [1155.5–1957.5]	1501.5 [1174–1924]	1546.0 [1194–1924]	0.350
Carbohydrates (g)^*α*^	54.0 [33.6–70.75]	46.3 [28.87–65.27]	44.6 [27.8–66.12]	48.6 [24.5–69.70]	0.707

^*α*^Values are expressed as median and quartiles. ^∗^The Kruskal-Wallis test was used to compare medians between BMI groups.

## Data Availability

Access to data is restricted in order to respect the rights of third parties and the confidentiality of participants.

## Abbreviations

NCD: Noncommunicable diseases
BMI: Body mass index
WC: Waist circumference
HC: Hip circumference
WHR: Waist-to-hip ratio
HCP: High commission for planning
SPSS: Statistical Package for the Social Sciences
EMRO: Eastern Mediterranean regional office.

## References

[1] World Health Organization (2018). *Factsheet: Malnutrition*.

[2] Institute of Medicine (2005). *Dietary, Reference Intakes for Energy, Carbohydrate, Fiber, Fat, Fattyacids, Cholesterol, Protein, and Amino Acids*.

[3] World Health Organization (2003). *Diet, Nutrition and the Prevention of Chronic Diseases*.

[4] Nutrition for Health and Development (NHD) (2000). *Sustainable Development, and Healthy Environment, (SDE), and World Health Organization (WHO), Turning the Tide of Malnutrition, Responding to the Chalenge of the 21st Century*.

[5] Baudin B. (2014). Malnutrition et sous-alimentation. *Revue Francophone des Laboratoires*.

[6] Latham M. C. (2001). *La nutrition dans les pays en développement*.

[7] The World Bank (2006). *Repositioning Nutrition as Central to Development. A Strategy for Large-Scale Action*.

[8] World Health Organization (2020). *Factsheet: Obesity and Overweight*.

[9] Collins K. H., Herzog W., Mac Donald G. Z. (2018). Obesity, metabolic syndrome, and musculoskeletal disease: common inflammatory pathways suggest a central role for loss of muscle integrity. *Frontiers in Physiology*.

[10] Chevallier L. (2009). *Nutrition: Principes et Conseils*.

[11] Prentice A. M. (2006). The emerging epidemic of obesity in developing countries. *International Journal of Epidemiology*.

[12] Fernald L. C., Gutierrez J. P., Neufeld L. M. (2004). High prevalence of obesity among the poor in Mexico. *Jama*.

[13] Ministère de la Santé (2018). *Enquête sur la Population et la Santé Familiale-2018*.

[14] Ministère de la Santé (2005). *Enquête sur la Population et la Santé Familiale 2003-2004*.

[15] Cochran W. G. (1977). *Sampling Techniques*.

[16] Ardilly P. (2006). *Les Techniques de Sondage*.

[17] World Health Organization (2000). Obesity: preventing and managing the global epidemic.

[18] National Institutes of Health (1998). National Heart Lung and Blood Institute, clinical guidelines on the identification, evaluation, and treatment of overweight and obesity in adults: the evidence report. *Obesity Research*.

[19] WHO (2008). *WHO STEP Wise Approach to Surveillance (STEPS)*.

[20] Dop M. C., Milan C., N’Diaye A. M, N’Diaye C. (1994). The 24-hour recall for Senegalese weanlings: a validation exercise. *European Journal of Clinical Nutrition*.

[21] Thompson E., Subar A. F. (2107). Dietary assessment methodology: chapter 1. *Nutrition in the Prevention and Treatment of Disease*.

[22] Stote K. S., Radecki S. V., Moshfegh A. J., Ingwersen L. A., Baer D. J. (2011). The number of 24 h dietary recalls using the US department of agriculture’s automated multiple-pass method required to estimate nutrient intake in overweight and obese adults. *Public Health Nutrition*.

[23] Herceberg S., Deheeger M., Preziosi P. (2002). Portions alimentaires: manuel photos pour l’estimation des quantités. *Broché*.

[24] Elmoumni K., Maimouni E., Dufourny G. (2008). *Haute Ecole Lucia de Brouck`ere-CIRIHA. Département de Diététique et Nutrition Appliquée*.

[25] Nutrilog, “Database”, 2016, http://www.nutrilog.com/nutrilog_fr/nutrilog_databases.htm

[26] Ghasem A., Zahediasl S. (2012). Normalitytestsforstatisticalanalysis:aguidefornon-statisticians. *International Journal of Endocrinology Metabolism*.

[27] Haut-Commissariat au Plan (2016). *Les Indicateurs Sociaux 2012-2013, édition 2016*.

[28] Jafri A., Jabari M., Dahhak M., Saile R., Derouiche A. (2013). Obesity and its related factors among women from popular neighborhoods in Casablanca, Morocco. *Ethnicity and Disease*.

[29] Rahim S., Baali A. (2011). Etude de l’obésité et quelques facteurs associés chez un groupe de femmes marocaines résidentes de la ville de Smara (sud du Maroc). *Antropo*.

[30] Gartner A., El Ati J., Traissac P. (2013). A double burden of overall or central adiposity and anemia or iron deficiency is prevalent but with little socioeconomic patterning among Moroccan and Tunisian urban women. *The Journal of Nutrition*.

[31] Barich F., Zahrou F. E., Laamiri F. Z. (2018). Association of obesity and socioeconomic status among women of childbearing age living in urban area of Morocco. *Journal of Nutrition and Metabolism*.

[32] Laamiri F. Z., Hasswane N., Kerbach A. (2016). Risk factors associated with a breast cancer in a population of Moroccan women whose age is less than 40 years: a case control stud. *Pan Africa in Medical Journal*.

[33] Ministère de la Santé (2016). *Stratégie Multisectorielle de Prévention et de Contrôle des Maladies Non Transmissibles 2016-2025*.

[34] WHO and Regional Office for the Eastern Mediterranean (2010). *Document Technique Stratégie Régionale sur la Nutrition 2010-2019*.

[35] FAO (2002). *Agriculture, Alimentation et Nutrition en Afrique: Un Ouvrage de référence à l’usage des Professeurs d’agriculture*.

[36] Dodd K. W., Guenther P. M., Freedman L. S. (2006). Statistical methods for estimating usual intake of nutrients and foods: a review of the theory. *Journal of the American Dietetic Association*.

[37] Forrestal S. G. (2011). Energy intake misreporting among children and adolescents: a literature review. *Maternal & Child Nutrition*.

[38] Maurer J., Taren D. L., Teixeira P. J. (2006). The psychosocial and behavioral characteristics related to energy misreporting. *Nutrition Reviews*.

[39] French Institute of Nutrition (1996). *Abords Méthodologiques des Enquêtes de Consommation Alimentaire Chez l’homme*.

[40] Dwyer J., Picciano M. F., Raiten D. J. (2003). Estimation of usual intakes: what we eat in America-NHANES. *The Journal of Nutrition*.

[41] Novotny J. A., Rumpler W. V., Riddick H. (2003). Personality characteristics as predictors of underreporting of energy intake on 24-hour dietary recall interviews. *Journal of the American Dietetic Association*.

[42] Klesges R. C., Eck L. H., Ray J. A. W. (1995). Who underreports dietary intake in a dietary recall? Evidence from the second national health and nutrition examination survey. *Journal of Consulting and Clinical Psychology*.

[43] Bassett M. N., Romaguera D., Samman N. (2011). Nutritional status and dietary habits of the population of the Calchaqui valleys of Tucuman, Argentina. *Nutrition*.

[44] Shim J. S., Oh K., Kim H. C. (2014). Dietary assessment methods in epidemiologic studies. *Epidemiology and Health*.

[45] Steyn N. P., Nel J. H., Parker W., Ayah R., Mbithe D. (2012). Urbanisation and the nutrition transition: a comparison of diet and weight status of South African and Kenyan women. *Scandinavian Journal of Public Health*.

[46] Zaghloul S., Al-Hooti S. N., Al-Hamad N. (2012). Evidence for nutrition transition in Kuwait: over-consumption of macronutrients and obesity. *Public Health Nutrition*.

[47] WHO and FAO (2003). Diet, nutrition and the prevention of chronic diseases, report of a Joint WHO/FAO expert consultation.

[48] WHO (2002). *Diet, Nutrition and the Prevention of Chronic Diseases*.

[49] Souza R. A. G., Yokoo E. M., Sichieri R., Pereira R. A. (2015). Energy and macronutrient intakes in Brazil: Results of the first nationwide individual dietary survey. *Public Health Nutrition*.

[50] Saizonou J., Jerome C. S., Maiga C., Kpozehouen A., Paraiso M. N., Ouendo E. M. D. (2017). Lesapportsalimentairesenferchezlafemmeenagerdeprocréerdanslacommuned’OuidaauBenin. *Revue marocaine de santé publique*.

[51] Afssa M. (2001). *Ambroise, Les Apports Nutritionnels de la Population Française*.

[52] Institute Of Medicine (2006). *Les Apports Nutritionnels de Référence (ANREF): Le Guide Essentiel des Besoins en Nutriments*.

[53] Direction du Ministère de l’intérieur et des Collectivités Locales (2015). *La Région de Rabat-Salé-Kenitra*.

[54] Allali F. (2017). Evolution des pratiques alimentaires au Maroc. *International Journal of Medicine and Surgery*.

[55] Te Morenga L. A., Howatson A. J., Jones R. M., Mann J. (2014). Dietary sugars and cardiometabolic risk: systematic review and meta-analyses of randomized controlled trials of the effects on blood pressure and lipids. *The American Journal of Clinical Nutrition*.

[56] Salter A. M. (2013). Dietary fatty acids and cardiovascular disease. *Animal*.

[57] Mensink R. P., Zock P. L., Kester A. D., Katan M. B. (2003). Effects of dietary fatty acids and carbohydrates on the ratio of serum total to HDL cholesterol and on serum lipids and apolipoproteins: a meta-analysis of 60 controlled trials. *The American Journal of Clinical Nutrition*.

[58] Fryar C. D., Wright J. D., Eberhardt M. S., Dye B. A. (2012). Trends in nutrient intakes and chronic health conditions among Mexican-American adults, a 25-year profile: United States 1982–2006. *National Health Statistics Reports*.

[59] Gopalan S. (2000). Malnutrition: causes, conséquences and solutions. *Nutrition*.

[60] Van de Poel E., Hosseinpoor A. R., Speybroeck N., Ourti T. V., Vega J. (2008). Socioeconomic inequality in malnutrition in developing countries. *Bulletin of the World Health Organization*.

[61] Ivanovitch K., Klaewkla J., Chongsuwat R., Viwatwongkasem C., Kitvorapat W. (2014). The intake of energy and selected nutrients by Thai urban sedentary workers: an evaluation of adherence to dietary recommendations. *Journal of Nutrition and Metabolism*.

[62] Jessri M., Lou W. Y., L’Abbé M. R. (2016). Evaluation of different methods to handle misreporting in obesity research: evidence from the Canadian national nutrition survey. *British Journal of Nutrition*.

[63] Romieu I., Dossus L., Dossus L. (2017). Energy balance and obesity: what are the main drivers?. *Cancer Causes & Control*.

[64] Organisation Mondiale de la Sante (2017). *obésité et surpoids les principaux faits*.

[65] Organisation Mondiale de la Sante (2017). *Suivi des Progrès 2017 Dans la Lutte Contre les Maladies Non Transmissibles*.

